# Insomnia in patients treated with checkpoint inhibitors for cancer: A meta-analysis

**DOI:** 10.3389/fonc.2022.946307

**Published:** 2022-08-02

**Authors:** Igor Kiss, Matyas Kuhn, Kristian Hrusak, Benjamin Buchler, Ludmila Boublikova, Tomas Buchler

**Affiliations:** ^1^ Department of Comprehensive Cancer Care, Masaryk Memorial Cancer Institute and Faculty of Medicine, Masaryk University, Brno, Czechia; ^2^ Institute of Biostatistics and Analyses, Masaryk University, Brno, Czechia; ^3^ Department of Oncology, First Faculty of Medicine, Charles University and Thomayer University Hospital, Prague, Czechia

**Keywords:** immunotherapy, cancer, checkpoint inhibitors, insomnia, systematic analysis

## Abstract

**Purpose:**

Insomnia in cancer patients is a common symptom contributing to poor quality of life and poor functioning. Sleep disturbances have been associated with inflammatory activity, and systemic cancer therapies chemotherapy, hormonal therapy, and immunotherapy may cause insomnia. We have carried out a meta-analysis to estimate the occurrence of insomnia in patients with solid cancer treated with immunotherapy using checkpoint inhibitors (CPI).

**Methods:**

PubMed and ClinicalTrials.gov were searched for phase 3 studies in solid tumours where treatment included a checkpoint inhibitor in the experimental arm. Data on the incidence of insomnia were acquired from the adverse events tables available from clinicaltrials.gov and/or from the full texts. Random effect logistic model was used to compare pooled data. Heterogeneity between studies was assessed using Cochrane Q statistics and I^2^ statistics.

**Results:**

A total of 54 studies (including six three-arm studies) involving 37,352 patients were included in the analysis. Insomnia was reported in 8.3% of subjects (95% confidence interval [CI] 8.0%-8.7%) treated with immunotherapy. Insomnia was significantly more common in patients receiving immunotherapy compared to those enrolled in study arms with inactive treatment (odds ratio [OR] 1.49, 95% CI 1.13-1.96). The odds for insomnia were similar between the arms for studies comparing CPI versus chemotherapy and CPI versus non-immunologic targeted therapies (OR 1.07, 95% CI 0.94-1.22 and OR 1.40, 95% CI 0.90-2.18, respectively). The OR for insomnia was higher for cytotoxic T-lymphocyte antigen 4 (CTLA-4) receptor inhibitors compared to the inhibitors of programmed death-1 (PD-1) receptor (OR 1.36, 95% CI 1.06 – 1.74).

**Conclusion:**

Cancer immunotherapy using CPI is associated with insomnia but the odds of developing the symptom are not greater with immunotherapy than with other systemic modalities including chemotherapy and non-immunologic targeted therapies.

## Introduction

Insomnia is a common and underestimated problem in cancer patients. Insomnia is an important contributing factor to poor quality of life, chronic fatigue, and impaired cognitive functioning. The aetiology of insomnia in cancer patients is multifactorial. Sleep disturbances may be the cause but also the consequence of chronic fatigue, depression, anxiety, and cognitive impairment. Important causes of insomnia in cancer patients also include pain or physical discomfort, decreased physical activity and changes in sleeping routine, such as occurring during hospitalizations (1).

Sleep disturbances have been linked to increased cancer risk, with evidence pointing to a causal relationship between lack of sleep and, especially, endocrine function-related cancers such as prostate and breast carcinoma (2–4). On the other hand, the chronic inflammatory state associated with conditions such as diabetes, autoimmune disease, and cancer has been shown to trigger disruption in circadian rhythm manifesting as insomnia (5–7). Systemic cancer treatments including chemotherapy, hormonal therapy, and immunotherapy using checkpoint inhibitors (CPI) have been associated with insomnia (8–10). CPIs are a part of standard treatment for many solid and haematological malignancies, radically improving the prognosis of a significant proportion of patients. However, monoclonal antibodies inhibiting the programmed death (PD)-1 receptor, its ligand PD-L1, and the cytotoxic T-lymphocyte antigen 4 (CTLA-4) receptor are also associated with activation of inflammatory processes. Treatment with these agents specifically designed to stimulate antitumour immune responses leads to complex changes in the immune system (11, 12). Due to the strong link between inflammation and sleep disorders, there is a rationale to examine the occurrence of insomnia during therapy with CPIs (13, 14).

The aim of the present meta-analysis was to examine the incidence of insomnia as an adverse event in clinical trials with CPIs in patients with solid cancers, and to compare its occurrence in patients treated with CPIs to those receiving other systemic therapies for solid cancers, including chemotherapy and non-immunologic targeted agents.

## Methods

### Study selection

**Figure 1:**
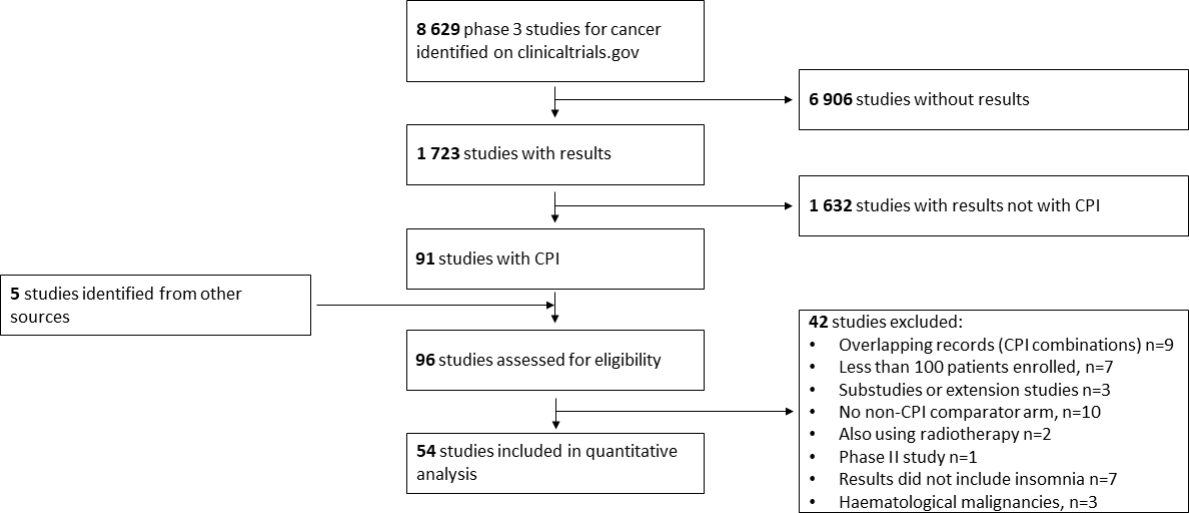
Selection process of the studies used in meta-analysis.

The search was carried out in the PubMed and ClinicalTrials.gov databases using terms “cancer” and “ipilimumab or MDX-010”, “nivolumab or MDX-1106”, “avelumab or MSB0010718C”, “durvalumab or MEDI-4736”, “pembrolizumab or MK-3475”, “atezolizumab or MPDL3280A”, “tremelimumab or CP-675,206”, “cemiplimab or REGN2810” (15). The database searches were performed on February 1, 2021. Furthermore, recent systematic studies were screened for further studies missed by the database search (16, 17). The study selection process is shown in **Figure 1**. The search was limited to phase 3 studies with *in extenso* publications in English and with tabulated adverse event data in the ClinicalTrials.gov database or in the available article. For all identified studies, the incidence of insomnia was determined from the adverse event tables. Two authors retrieved the data independently. The study was performed according to the Preferred Reporting Items for Systematic Reviews and Meta-analyses (PRISMA) guidelines (18).

### Statistical analysis

The percentages and confidence intervals of patients with insomnia were reported within each study, as well as an aggregate for the different classes of CPI agents. The odds ratio (OR) and confidence interval (CI) for each study were reported. The types of treatment in the CPI arms were classified as follows: CPI, CPI in combination with chemotherapy, and CPI in combination with non-immunologic targeted therapy. Differences between the individual types of CPI were analysed for the following categories: anti-PD-1 agents, anti-PDL-1 agents, anti-CTLA-4 agents, and combinations of anti-CTLA-4 agents with anti-PD-1/L1 antibodies (anti-PD-1 and anti-PD-L1 agents were analysed jointly in combinations with antiCTLA-4 drugs) (15). If control arm contained the combination of chemotherapy and a non-immunologic targeted agent, it was classified as "chemotherapy" for the meta-analysis.

The random effect model was used to compare pooled data (19). Two-arm and three-arm studies were included in the meta-analysis. A three-arm study with two experimental arms (E1 and E2) and one control arm (C) will generate two study arm pairs (E1 versus C; E2 versus C). Data from three-arm studies included in the meta-analysis were processed according to a method recommended by Rucker et al. (splitting the shared group of multi-arm trials in pairwise meta-analysis) (20).

Cochrane Q statistics and I^2^ statistics were used to estimate heterogeneity. Certainty of evidence was assessed per Grading of Recommendations, Assessment, Development and Evaluations (GRADE) guidelines (21). I^2^ values were used to classify heterogeneity as low (<25%), intermediate (25-75%), or high (>75%) (22).

The logistic model with random effect was used to compare different classes of immunotherapy agents, i.e. those targeting PD-1, PD-L1, and CTLA-4, respectively. All statistical analyses were performed using software R version 3.6.3 (R Foundation for Statistical Computing, Vienna, Austria) using the R package meta (23).

## Results

### Selection of studies

A total of 8,632 records of phase 3 studies for cancer were identified in the initial step of the search. Of 93 studies using CPI therapy in the experimental arm, 54 studies (including six three-arm studies) enrolling 37,352 patients with evaluated toxicity were included in the present analysis. The list of the included studies is provided in **Supplementary Table 1** (24–77).

The solid cancers treated in the included studies were the following: lung cancer (23 studies), melanoma (six studies), renal cancer (five studies), urothelial cancer (five studies), head and neck carcinoma (four studies), breast cancer (three studies), gastro-oesophageal junction cancer (three studies), mesothelioma (two studies), prostate cancer (two studies), gastric, oesophageal or colorectal cancer, hepatocellular carcinoma (one study each). The pairwise analysis was carried out comparing 60 study arm-pairs: two study pairs were generated for each of the three-arm studies comparing each of the CPI-containing arms with the control arm. Because high-grade (grade 3) insomnia was not reported in the included studies, all-grade insomnia was analysed (78). Summary of the results is shown in **Table 1**.

**Table 1:** Risk of all-grade insomnia – summary of results.

Type of analysed studies	Arms	Number of participants	Number of study arm pairs	Rate of events, %(95% CI)	Odds ratio(95% CI)	Heterogeneity		Certainty of evidence
						Q (p value)	I2,% (95% CI)	
All	CPI	21192	60	8.3 (8.0–8.7)	**1.15 (1.05–1.25)**	73.4 (0.099)	19.6 (0.0–42.1)	High
	control	16160		7.4 (7.0–7.8)			
CPI vs inactive control	CPI	3431	10	7.9 (7.0–8.9)	**1.49 (1.13–1.96)**	13.6 (0.136)	33.9 (0.0–68.5)	Moderate
	control	2484		5.4 (4.5–6.3)			
CPI vs CT	CPI	8715	26	7.1 (6.6–7.7)	1.07 (0.94–1.22)	24.6 (0.483)	0.0 (0.0–42.4)	High
	control	6918		6.6 (6.0–7.2)			
CPI+CT vs CT	CPI	5851	16	10.3 (9.6–11.2)	1.13 (0.96–1.33)	21.6 (0.119)	30.5 (0.0–61.9)	Moderate
	control	4704		9.4 (8.6–10.3)			
CPI + TT vs TT	CPI	1328	3	9.9 (8.4–11.7)	1.40 (0.90–2.18)	4.9 (0.087)	59.0 (0.0–88.3)	Moderate
	control	1300		7.3 (6.0–8.9)			

CPI, checkpoint inhibitor; CT, chemotherapy; TT, non-immunologic targeted therapy; CI, confidence interval.

Statistically significant differences between arms per odds ratio are highlighted.

The Cochrane risk of bias tool was used for quality assessment. The main source of was performance bias, i.e., the lack of blinding of participants and personnel in some studies (**Supplementary Table 1**). Because the analysed studies were all randomised phase III trials, there was a low risk of other types of bias including random sequence generation, allocation concealment, blinding of outcome assessment, incomplete outcome data, and selective reporting bias. The risk of evidence selection bias was low because insomnia was not the main assessed parameter or the clinically most important toxicity in any of the studies.

### Overall incidence of insomnia in patients treated with CPI

Insomnia was reported in 8.3% of subjects (95% CI 8.0%-8.7%) treated with immunotherapy. Across all types of control arms, the OR of insomnia was 1.15 (95% CI 1.05–1.25) (**Table 2**). The highest OR for insomnia was detected for the combination of antiCTLA-4 and antiPD-1/L1 agents (OR 1.36, 95% CI 1.06 – 1.75 using antiPD-1 agents as reference). The OR was also higher for antiCTLA-4 therapy compared to antiPD1 drugs (OR 1.36, 95% CI 1.05-1.74) and, moderately but statistically significantly also for antiPDL1 agents compared to antiPD1 drugs (OR 1.22, 95% CI 1.00-1.49) (**Table 3**). The heterogeneity was low for all drug classes except for the comparison of antiCTLA-4 versus antiPD-1 where it was intermediate (**Table 4**).

**Table 2:** Odds ratio of insomnia in randomised studies of checkpoint inhibitors versus all types of control (control arm as reference).

Study	Diagnosis	Inhibitor	N (contr/CPI)	OR (95% CI)		p value
**Hodi et al.,** (24)	**Melanoma**	CTLA-4	132/511	0.83 (0.45–1.53)	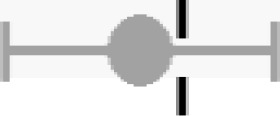	0.544
**Robert et al.,** (25)	**Melanoma**	CTLA-4	251/247	1.44 (0.74–2.80)	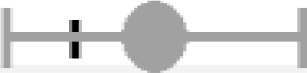	0.289
**Kwon et al.,** (36)	**Prostate**	CTLA-4	396/393	0.91 (0.55–1.52)	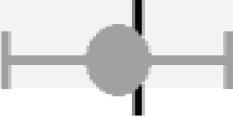	0.722
**Borghaei et al.,** (47)	**Lung**	PD-1	268/287	0.84 (0.45–1.57)	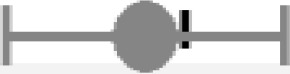	0.581
**Brahmer et al.,** (58)	**Lung**	PD-1	129/131	1.16 (0.38–3.54)	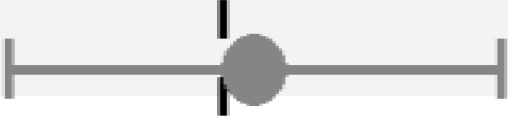	0.798
**Motzer et al.,** (69)	**Renal**	PD-1	397/406	1.06 (0.61–1.84)	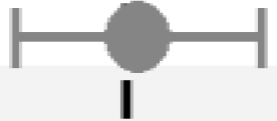	0.844
**Eggermont et al.,** (74)	**Melanoma**	CTLA-4	474/471	2.28 (1.34–3.89)	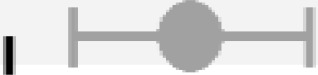	0.003
**Ferris et al.,** (75)	**Head and neck**	PD-1	111/236	0.80 (0.30–2.08)	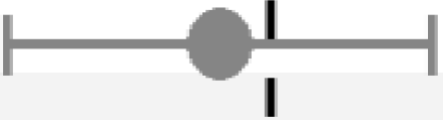	0.641
**Herbst et al.,** (76)	**Lung**	PD-1	309/682	0.95 (0.55–1.64)	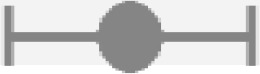	0.85
**Reck et al.,** (77 **), 1**	**Lung**	CTLA-4	561/562	0.84 (0.57–1.23)	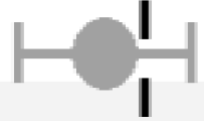	0.371
**Reck et al.,** (26 **), 2**	**Lung**	PD-1	150/154	1.44 (0.60–3.49)	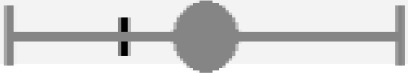	0.414
**Antonia et al.,** (27)	**Lung**	PD-L1	234/475	1.34 (0.75–2.39)	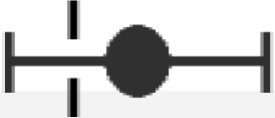	0.329
**Beer et al.,** (28)	**Prostate**	CTLA-4	199/399	2.22 (1.05–4.69)	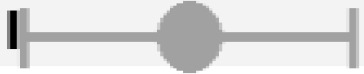	0.036
**Bellmunt et al.,** (29)	**Urothelial**	PD-1	255/266	0.79 (0.40–1.58)	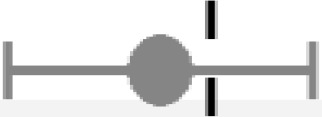	0.514
**Carbone et al.,** (30)	**Lung**	PD-1	263/267	0.87 (0.44–1.72)	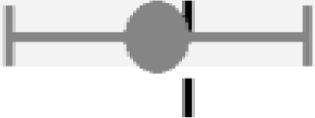	0.695
**Govindan et al., 2017** (79)	**Lung**	CTLA-4	473/475	1.27 (0.85–1.90)	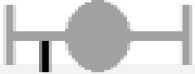	0.234
**Maio et al., 2017** (80)	**Mesothelioma**	CTLA-4	189/380	1.12 (0.57–2.21)	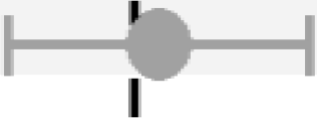	0.746
**Rittmeyer et al.,** (32)	**Lung**	PD-L1	578/609	1.26 (0.83–1.91)	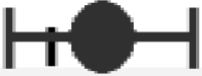	0.275
**Barlesi et al.,** (78)	**Lung**	PD-L1	365/393	0.79 (0.42–1.49)	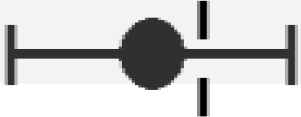	0.469
**Gandhi et al.,** (34)	**Lung**	PD-1	202/405	0.85 (0.43–1.67)	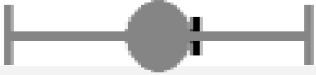	0.63
**Horn et al.,** (35)	**Lung**	PD-L1	196/198	1.15 (0.53–2.49)	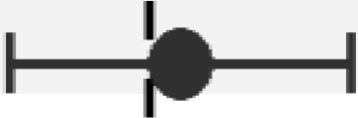	0.716
**Larkin et al.,** (37)	**Melanoma**	PD-1	102/268	2.17 (0.88–5.36)	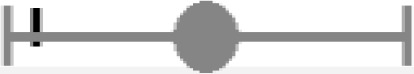	0.093
**Motzer et al.,** (38)	**Renal**	CTLA-4+PD-1	535/547	1.69 (1.09–2.62)	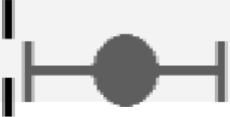	0.018
**Paz-Ares et al.,** (39)	**Lung**	PD-1	280/278	1.25 (0.70–2.23)	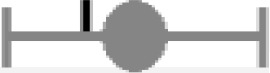	0.447
**Powles et al.,** (40)	**Urothelial**	PD-L1	443/459	1.04 (0.67–1.62)	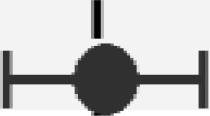	0.869
**Shitara et al.,** (41)	**Gastric**	PD-1	276/294	0.60 (0.31–1.16)	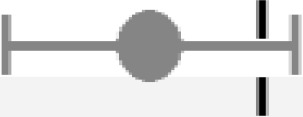	0.132
**Schmid et al.,** (42)	**Breast**	PD-L1	430/460	0.97 (0.64–1.45)	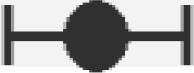	0.871
**Socinski et al.,** (33)	**Lung**	PD-L1	394/793	1.21 (0.81–1.81)	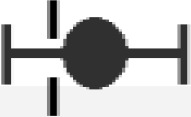	0.34
**Cohen et al.,** (43)	**Head and neck**	PD-1	234/246	1.25 (0.65–2.43)	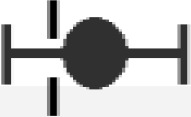	0.502
**Eng et al.,** (44)	**Colorectal**	PD-L1	80/269	1.52 (0.43–5.37)	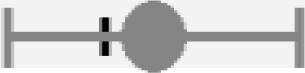	0.519
**Mok et al.,** (45)	**Lung**	PD-1	615/635	0.71 (0.45–1.13)	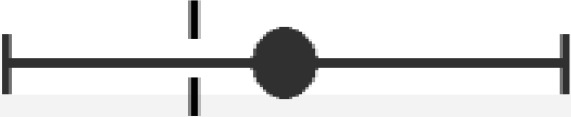	0.152
**Paz-Ares et al.,** (73)	**Lung**	CTLA-4+PD-1	266/266	1.93 (0.96–3.88)	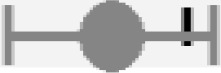	0.065
**Paz-Ares et al.,** (73)	**Lung**	PD-L1	266/265	1.85 (0.92–3.73)	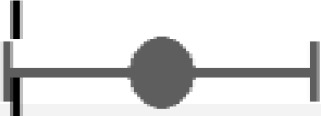	0.086
**Rini, Plimack, et al.,** (48)	**Renal**	PD-1	425/429	0.91 (0.56–1.46)	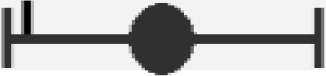	0.685
**Rini, Powles, et al.,** (49)	**Renal**	PD-L1	446/451	0.99 (0.61–1.61)	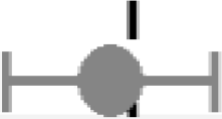	0.961
**West et al.,** (50)	**Lung**	PD-L1	232/473	1.09 (0.69–1.72)	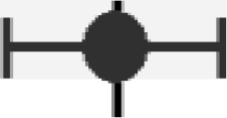	0.716
**Wu et al.,** (68)	**Lung**	PD-1	156/337	0.73 (0.36–1.50)	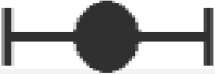	0.393
**Burtness et al.,** (70)	**Head and neck**	PD-1	287/276	1.24 (0.70–2.19)	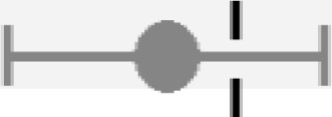	0.466
**Ferris et al.,** (51)	**Head and neck**	CTLA-4+PD-1	240/246	1.62 (0.66–3.98)	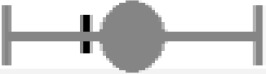	0.294
**Ferris et al.,** **(** 51)	**Head and neck**	PD-L1	240/237	0.88 (0.31–2.47)	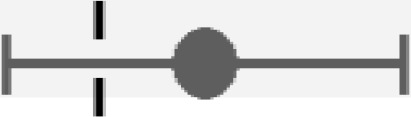	0.812
**Finn et al.,** (71)	**HCC**	PD-1	134/279	0.71 (0.28–1.78)	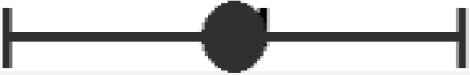	0.461
**Gutzmer et al.,** (52)	**Melanoma**	PD-L1	281/230	0.76 (0.36–1.60)	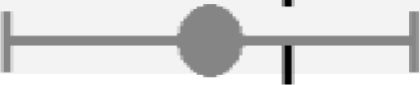	0.468
**Herbst et al.,** (53)	**Lung**	PD-L1	263/286	1.38 (0.70–2.72)	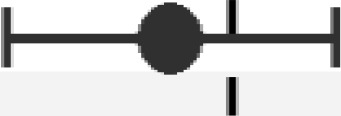	0.355
**Jotte et al.,** (54)	**Lung**	PD-L1	334/334	1.12 (0.65–1.93)	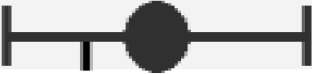	0.678
**Kojima et al.,** (55)	**Esophagus**	PD-1	296/314	1.51 (0.79–2.90)	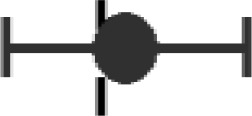	0.21
**Mittendorf et al.,** (56)	**Breast**	PD-L1	164/167	0.56 (0.33–0.95)	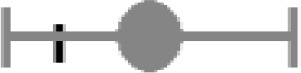	0.03
**Powles et al.,** (57)	**Urothelial**	CTLA-4+PD-1	315/340	1.71 (0.87–3.34)	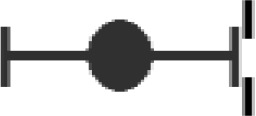	0.12
**Powles et al.,** (57)	**Urothelial**	PD-L1	315/345	1.68 (0.86–3.29)	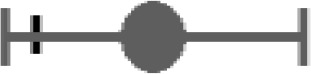	0.131
**Powles et al.,** (59 **)**	**Urothelial**	PD-L1	345/344	2.74 (1.20–6.27)	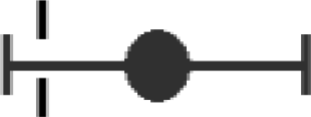	0.017
**Rizvi et al.,** (60)	**Lung**	PD-L1	352/369	1.18 (0.66–2.12)	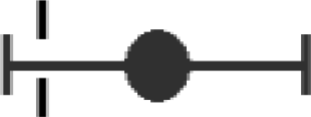	0.57
**Rizvi et al.,** (60)	**Lung**	CTLA-4+PD-1	352/371	1.66 (0.96–2.88)	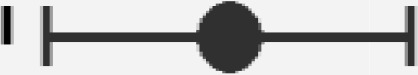	0.07
**Rudin et al.,** (61)	**Lung**	PD-1	223/223	0.88 (0.50–1.56)	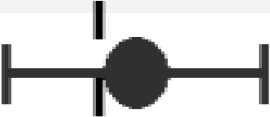	0.661
**Baas et al.,** (62)	**Mesothelioma**	CTLA-4	284/300	1.77 (0.92–3.41)	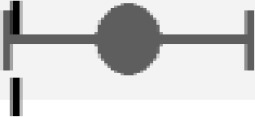	0.086
**Motzer et al.,** (63)	**Renal**	PD-1	340/352	1.84 (1.06–3.20)	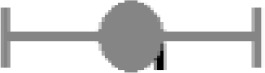	0.032
**Owonikoko et al.,** (64)	**Lung**	PD-1	273/279	1.12 (0.55–2.28)	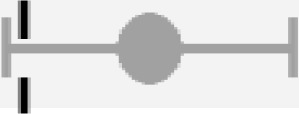	0.764
**Owonikoko et al.,** (64)	**Lung**	CTLA-4+PD-1	273/165	2.37 (1.18–4.78)	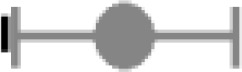	0.016
**Paz-Ares et al., 2021**	**Lung**	CTLA-4+PD-1	349/358	1.84 (0.90–3.78)	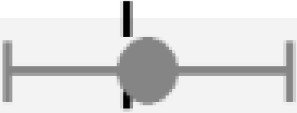	0.097
**Powles et al.,** (65)	**Urothelial**	PD-1	342/349	2.27 (1.23–4.19)	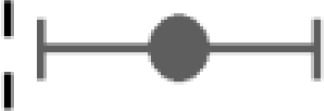	0.009
**Powles et al.,** (65)	**Urothelial**	PD-1	342/302	1.37 (0.69–2.71)	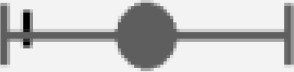	0.369
**Winer et al.,** (66)	**Breast**	PD-1	292/309	0.55 (0.24–1.29)	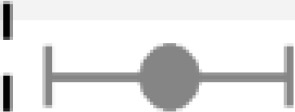	0.169
**Total: antiCTLA-4**		CTLA-4	2959/3738	1.27 (0.98–1.64)	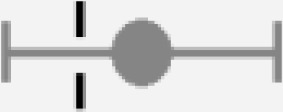	0.074
**Total: antiPD-1**		PD-1	6359/8004	1.02 (0.89–1.17)	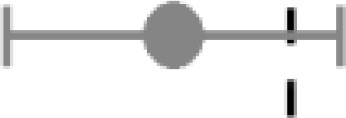	0.777
**Total: antiCTLA-4+antiPD-1**		CTLA-4+PD-1	2330/2293	1.79 (1.42–2.27)	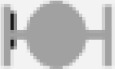	<0.001
**Total: antiPD-L1**		PD-L1	5958/7157	1.11 (0.97–1.27)	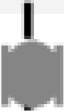	0.118
**Total**			16160/21192	1.15 (1.05–1.25)	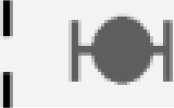	0.003

Contr, control arm; CPI, checkpoint inhibitor arm; OR, odds ratio; CI, confidence interval; HCC, hepatocellular carcinoma; PD-1, programmed death-1; PD-L1, programmed death-1 ligand; CTLA-4, cytotoxic T-lymphocyte antigen 4.

**Table 3:** Odds ratio of insomnia according to type of immunotherapy (antiPD-1 agents as reference).

Study arm	Receptor (immunotherapy arm)	OR (95% CI)		p value
**Total**	**CTLA-4**	**1.24 (0.96–1.60)**	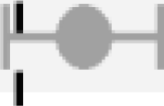	0.101
	**CTLA-4+PD-1**	**1.21 (0.94–1.54)**	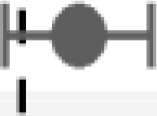	0.135
	**PD-L1**	**1.16 (0.95–1.42)**	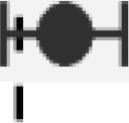	0.136
**Control arm**	**CTLA-4**	**1.08 (0.78–1.48)**	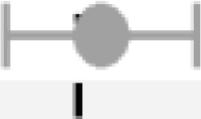	0.642
	**CTLA-4+PD-1**	**0.80 (0.55–1.16)**	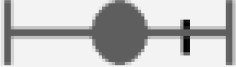	0.239
	**PD-L1**	**1.11 (0.87–1.44)**	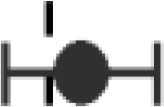	0.402
**Immunotherapy**	**CTLA-4**	**1.36 (1.06–1.74)**	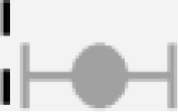	0.014
	**CTLA-4+PD-1**	**1.36 (1.05–1.75)**	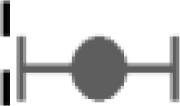	0.018
	**PD-L1**	**1.22 (1.00–1.49)**	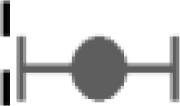	0.045

Contr, control arm; CPI, checkpoint inhibitor arm; OR, odds ratio; CI, confidence interval; PD-1, programmed death-1; PD-L1, programmed death-1 ligand; CTLA-4, cytotoxic T-lymphocyte antigen 4.

**Table 4:** Heterogeneity estimates for all studies/study arms.

Group	Number of studies	N (contr/CPI)	Q (p value)	I2 (95% CI) [%]
**Total: CTLA-4**	9	2959/3738	15.7 (0.046)	49.2 (0.0–76.3)
**Total: PD-1**	25	6359/8004	25.2 (0.394)	4.9 (0.0–35.9)
**Total: CTLA-4+PD-1**	7	2330/2293	0.9 (0.990)	0.0 (0.0–0.0)
**Total: PD-L1**	19	5958/7157	19.3 (0.375)	6.6 (0.0–40.9)
**Total**	60	16160/21192	73.4 (0.099)	19.6 (0.0–42.1)

Contr, control arm; CPI, checkpoint inhibitor arm; CI, confidence interval; PD-1, programmed death-1; PD-L1, programmed death-1 ligand; CTLA-4, cytotoxic T-lymphocyte antigen 4.

### CPI versus inactive control arm (placebo and/or best supportive care)

Nine studies were carried out comparing a CPI to inactive treatment, including one three-arm study. In total, 10 study arm pairs were analysed. The control arm was considered inactive if the allocated patients received placebo therapy or best supportive care but not active antineoplastic systemic agents. Insomnia was significantly more common in patients receiving immunotherapy compared to those enrolled in study arms with inactive treatment (OR 1.49, 95% CI 1.13-1.96). There was an intermediate heterogeneity among the studies (**Table 5**).

**Table 5:** Odds ratio of insomnia in randomised studies of checkpoint inhibitors versus inactive treatment (placebo and/or best supportive care), with control arm used as reference.

Study	Diagnosis	Inhibitor	N (contr/CPI)	OR (95% CI)		p value
**Kwon et al.,** (36)	**Prostate**	CTLA-4	396/393	0.91 (0.55–1.52)	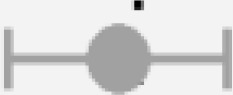	0.722
**Eggermont et al.,** (74)	**Melanoma**	CTLA-4	474/471	2.28 (1.34–3.89)	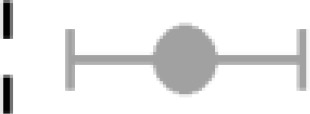	0.003
**Antonia et al.,** (27)	**Lung**	PD-L1	234/475	1.34 (0.75–2.39)	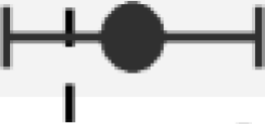	0.329
**Beer et al.,** (28)	**Prostate**	CTLA-4	199/399	2.22 (1.05–4.69)	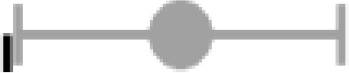	0.036
**Maio et al., ** (80)	**Mesothelioma**	CTLA-4	189/380	1.12 (0.57–2.21)	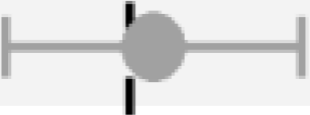	0.746
**Ferris et al.,** (51)	**Head and neck**	CTLA-4+PD-1	240/246	1.62 (0.66–3.98)	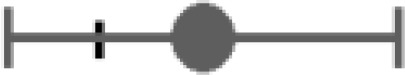	0.294
**Finn et al.,** (71)	**HCC**	PD-1	134/279	0.71 (0.28–1.78)	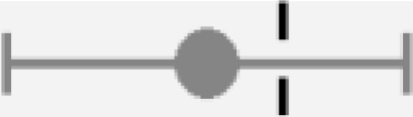	0.461
**Powles et al.,** (59 **)**	**Urothelial**	PD-L1	345/344	2.74 (1.20–6.27)	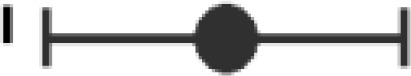	0.017
**Owonikoko et al.,** (64)	**Lung**	PD-1	273/279	1.12 (0.55–2.28)	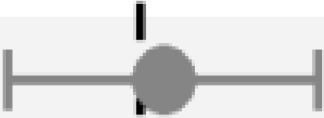	0.764
**Owonikoko et al.,** (64)	**Lung**	CTLA-4+PD-1	273/165	2.37 (1.18–4.78)	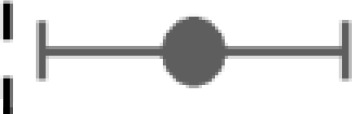	0.016
**Total**			2484/3431	1.49 (1.13–1.96)	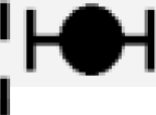	0.005

Contr, control arm; CPI, checkpoint inhibitor arm; OR, odds ratio; CI, confidence interval; HCC, hepatocellular carcinoma; PD-1, programmed death-1; PD-L1, programmed death-1 ligand; CTLA-4, cytotoxic T-lymphocyte antigen 4.

### CPI versus chemotherapy

The meta-analysis was carried out for 24 individual randomised studies including two three-arm studies. The odds for insomnia were similar between the arms (OR 1.07, 95% CI 0.94-1.22). There was a low heterogeneity among the studies (**Table 6**).

**Table 6:** Odds ratio of insomnia in randomised studies of checkpoint inhibitors versus chemotherapy (control arm as reference).

Study	Diagnosis	Receptor	N (contr/CPI)	OR (95% CI)		p value
**Borghaei et al.,** (47)	**Lung**	PD-1	268/287	0.84 (0.45–1.57)	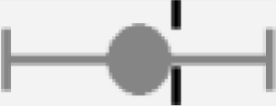	0.581
**Brahmer et al.,** (58)	**Lung**	PD-1	129/131	1.16 (0.38–3.54)	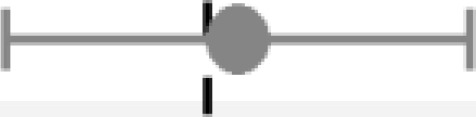	0.798
**Ferris et al.,** (75)	**Head and neck**	PD-1	111/236	0.80 (0.30–2.08)	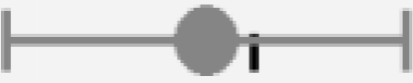	0.641
**Herbst et al.,** (76)	**Lung**	PD-1	309/682	0.95 (0.55–1.64)	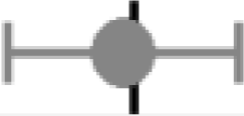	0.850
**Reck et al.,** (26 **)**	**Lung**	PD-1	150/154	1.44 (0.60–3.49)	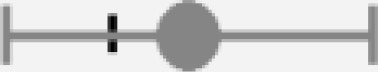	0.414
**Bellmunt et al.,** (29)	**Urothelial**	PD-1	255/266	0.79 (0.40–1.58)	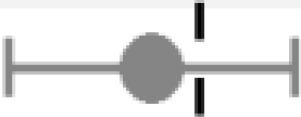	0.514
**Carbone et al.,** (30)	**Lung**	PD-1	263/267	0.87 (0.44–1.72)	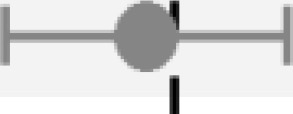	0.695
**Rittmeyer et al.,** (32)	**Lung**	PD-L1	578/609	1.26 (0.83–1.91)	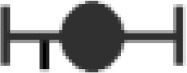	0.275
**Barlesi et al.,** (72)	**Lung**	PD-L1	365/393	0.79 (0.42–1.49)	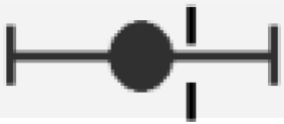	0.469
**Larkin et al.,** (37)	**Melanoma**	PD-1	102/268	2.17 (0.88–5.36)	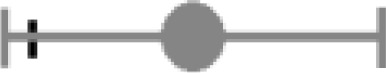	0.093
**Paz-Ares et al.,** (39)	**Lung**	PD-1	280/278	1.25 (0.70–2.23)	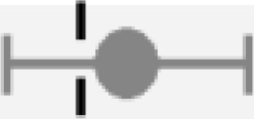	0.447
**Powles et al.,** (40)	**Urothelial**	PD-L1	443/459	1.04 (0.67–1.62)	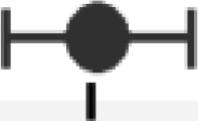	0.869
**Shitara et al.,** (41)	**Gastric**	PD-1	276/294	0.60 (0.31–1.16)	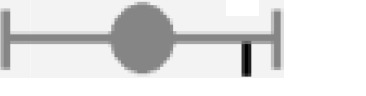	0.132
**Cohen et al.,** (43)	**Head and neck**	PD-1	234/246	1.25 (0.65–2.43)	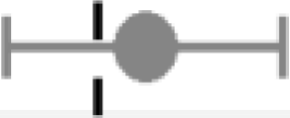	0.502
**Mok et al.,** (45)	**Lung**	PD-1	615/635	0.71 (0.45–1.13)	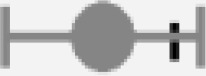	0.152
**Wu et al.,** (68)	**Lung**	PD-1	156/337	0.73 (0.36–1.50)	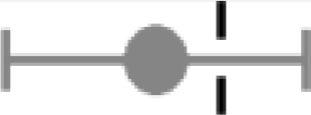	0.393
**Ferris et al.,** (51)	**Head and neck**	PD-L1	240/237	0.88 (0.31–2.47)	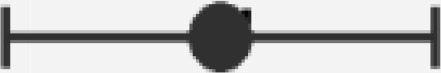	0.812
**Herbst et al.,** (53)	**Lung**	PD-L1	263/286	1.38 (0.70–2.72)	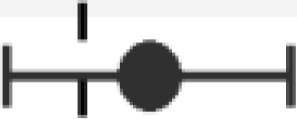	0.355
**Kojima et al.,** (55)	**Esophagus**	PD-1	296/314	1.51 (0.79–2.90)	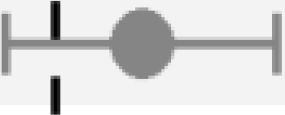	0.210
**Powles et al.,** (57)	**Urothelial**	CTLA-4+PD-1	315/340	1.71 (0.87–3.34)	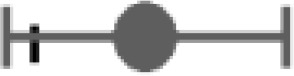	0.120
**Powles et al.,** (57)	**Urothelial**	PD-L1	315/345	1.68 (0.86–3.29)	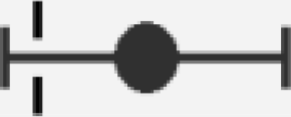	0.131
**Rizvi et al.,** (60)	**Lung**	PD-L1	352/369	1.18 (0.66–2.12)	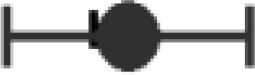	0.570
**Rizvi et al.,** (60)	**Lung**	CTLA-4+PD-1	352/371	1.66 (0.96–2.88)	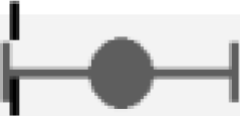	0.070
**Baas et al.,** (62)	**Mesothelioma**	CTLA-4	284/300	1.77 (0.92–3.41)	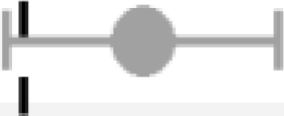	0.086
**Powles et al.,** (65)	**Urothelial**	PD-1	342/302	1.37 (0.69–2.71)	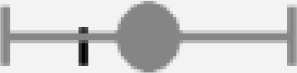	0.369
**Winer et al.,** (66)	**Breast**	PD-1	292/309	0.55 (0.24–1.29)	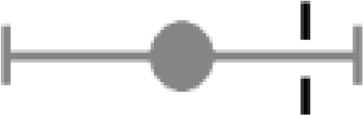	0.169
**Total**			6918/8715	1.07 (0.94–1.22)	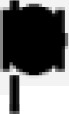	0.288

Contr, control arm; CPI, checkpoint inhibitor arm; OR, odds ratio; CI, confidence interval; PD-1, programmed death-1; PD-L1, programmed death-1 ligand; CTLA-4, cytotoxic T-lymphocyte antigen 4.

### CPI with non-immunologic targeted therapy versus non-immunologic targeted therapy alone

All studies (n=3) in this category involved therapy for metastatic renal cell carcinoma. There was a trend to increased occurrence of insomnia in the immunotherapy arms (OR 1.40, 95% CI 0.90-2.18) that however failed to reach statistical significance. There was an intermediate heterogeneity among the studies (**Table 7**).

**Table 7:** Odds ratio of insomnia in randomised studies of checkpoint inhibitors versus non-immunologic targeted therapies (control arm as reference).

Study	Diagnosis	Receptor	N (contr/CPI)	OR (95% CI)		p value
**Motzer et al.,** (38)	**Renal**	CTLA-4+PD-1	535/547	1.69 (1.09–2.62)	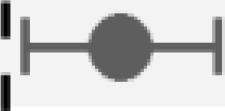	0.018
**Rini et al.,** (49)	**Renal**	PD-1	425/429	0.91 (0.56–1.46)	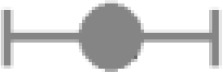	0.685
**Motzer et al.,** (63)	**Renal**	PD-1	340/352	1.84 (1.06–3.20)	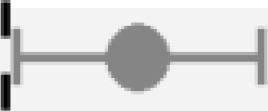	0.032
**Total**			1300/1328	1.40 (0.90–2.18)	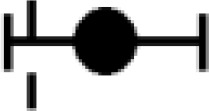	0.131

Contr, control arm; CPI, checkpoint inhibitor arm; OR, odds ratio; CI, confidence interval; PD-1, programmed death-1; PD-L1, programmed death-1 ligand; CTLA-4, cytotoxic T-lymphocyte antigen 4.

### CPI with chemotherapy versus chemotherapy alone

Fifteen studies including one three-arm study (i.e. 16 study arm pairs) were included in the analysis, of those 10 (66%) were carried out in lung cancer. There was no significant difference in the risk of insomnia (OR 1.13, 95% CI 0.96-1.33) with an intermediate heterogeneity (**Table 8**).

**Table 8:** Odds ratio of insomnia in randomised studies of checkpoint inhibitors combined with chemotherapy versus chemotherapy (control arm as reference).

Study	Diagnosis	Inhibitor	N (contr/CPI)	OR (95% CI)		p value
**Robert et al.,** (25)	**Melanoma**	CTLA-4	251/247	1.44 (0.74–2.80)	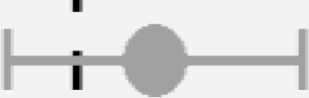	0.289
**Reck et al.,** (77 **)**	**Lung**	CTLA-4	561/562	0.84 (0.57–1.23)	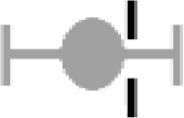	0.371
**Govindan et al.,** (79)	**Lung**	CTLA-4	473/475	1.27 (0.85–1.90)	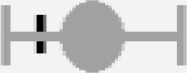	0.234
**Gandhi et al.,** (34)	**Lung**	PD-1	202/405	0.85 (0.43–1.67)	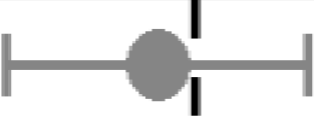	0.630
**Horn et al.,** (35)	**Lung**	PD-L1	196/198	1.15 (0.53–2.49)	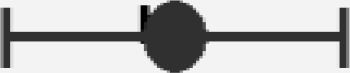	0.716
**Schmid et al.,** (42)	**Breast**	PD-L1	430/460	0.97 (0.64–1.45)	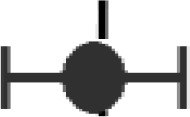	0.871
**Socinski et al.,** (33)	**Lung**	PD-L1	394/793	1.21 (0.81–1.81)	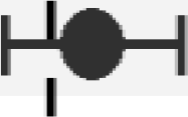	0.340
**Paz-Ares et al.,** (73)	**Lung**	CTLA-4+PD-1	266/266	1.93 (0.96–3.88)	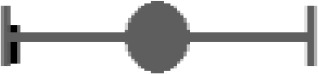	0.065
**Paz-Ares et al.,** (73)	**Lung**	PD-L1	266/265	1.85 (0.92–3.73)	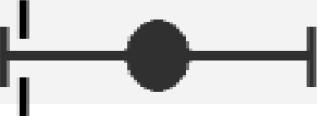	0.086
**West et al.,** (50)	**Lung**	PD-L1	232/473	1.09 (0.69–1.72)	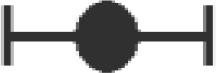	0.716
**Burtness et al.,** (70)	**Head and neck**	PD-1	287/276	1.24 (0.70–2.19)	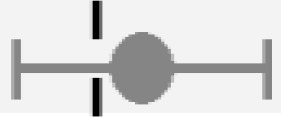	0.466
**Jotte et al.,** (54)	**Lung**	PD-L1	334/334	1.12 (0.65–1.93)	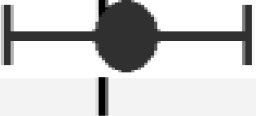	0.678
**Mittendorf et al.,** (56)	**Breast**	PD-L1	164/167	0.56 (0.33–0.95)	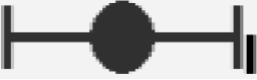	0.030
**Rudin et al.,** (61)	**Lung**	PD-1	223/223	0.88 (0.50–1.56)	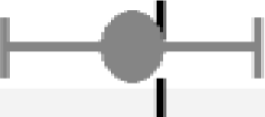	0.661
**Paz-Ares et al.,** (49)	**Lung**	CTLA-4+PD-1	349/358	1.84 (0.90–3.78)	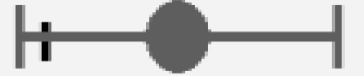	0.097
**Powles et al.,** (65)	**Urothelial**	PD-1	342/349	2.27 (1.23–4.19)	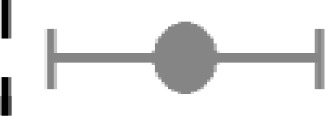	0.009
**Total**			4704/5851	1.13 (0.96–1.33)	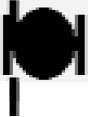	0.143

Contr, control arm; CPI, checkpoint inhibitor arm; OR, odds ratio; CI, confidence interval; PD-1, programmed death-1; PD-L1, programmed death-1 ligand; CTLA-4, cytotoxic T-lymphocyte antigen 4.

## Discussion

The results of the present extensive meta-analysis of phase III trials indicate that treatment with CPI for solid cancers is associated with increased risk of insomnia. However, the odds of insomnia are not increased compared to other systemic antineoplastic modalities such as chemotherapy and non-immunologic targeted therapies. Immune system activation occurring with CPI therapy is the putative causative mechanism linking the treatment with insomnia.

There is a strong, bidirectional link between insomnia and inflammation. Poor sleeping consistency has been associated with increase in inflammatory markers, including interleukin (IL)-6 and C-reactive protein (CRP) as well as serum amyloid-α, tumour necrosis factor-α, and granulocyte-macrophage colony-stimulating factor (13, 14). IL-6 is a proinflammatory cytokine and elevated levels have been detected in advanced cancer as well as during autoimmune adverse events in patients treated with CPI. Indeed, an anti-IL-6 agent, tocilizumab, is used for the treatment of corticosteroid-refractory autoimmune toxicities (81–83). IL-17 has also been associated with CPI efficacy and toxicity but also with sleep restriction (84–86).

Evidence suggests a common link between circadian cycle and cancer mediated by circadian core genes (2–4). An extensive meta-analysis of related to sleep disorders and inflammatory markers confirmed association between disturbed circadian rhythms and inflammatory markers including CRP and IL-6 (13). The circadian rhythm of IL-6 is altered in patients with chronic insomnia, providing a possible link between chronic inflammatory state induced by cancer and/or CPI and insomnia (87). Fatigue is another result of this immune activation, and the question has been addressed in a recent analysis by our group (15).

Recently, in an animal model, overexpression of NF-kB has been identified as the common underlying factor for insomnia and inflammation (88). Circadian clock genes play a complex role in cancer development and anti-cancer immune response, regulating even the formation of tumour-related immune cell infiltrates (88, 89). Thus, there is ample evidence that excessive, chronic inflammatory activation may provide a link between cancer, cancer therapies, and insomnia (5, 90).

Interestingly, on the other side of the spectrum of sleep disorders, narcolepsy type 1 is thought to have autoimmune aetiology and T cells directed against hypocretin/orexin neurons have been identified in some patients (91). A case report has been published of narcolepsy possibly caused by pembrolizumab (92).

A recent pioneering study, the first to look specifically at the population of cancer patients treated with CPI has been published by (10). They did not find any association between the occurrence of insomnia, obstructive sleep apnoea and the number of CPI infusions. However, the study was relatively small and, as our analysis shows, the effect of CPI on insomnia is relatively modest.

Insomnia recorded during a cancer-related clinical trial is self-reported and is a composite endpoint covering sleep inconsistency (night-to-night variability in sleep pattern), short sleep duration relative to patient previous habits or expectations, poor sleep quality (including mid-sleep awakenings), and unrefreshing sleep. Insomnia as an adverse event represents an increase in the severity of the symptom over the study period and the follow-up. Thus, the relatively low incidence of insomnia in the analysed studies does not reflect the pre-existing insomnia which is thought to affect 30-75% of cancer patients, a prevalence approximately three times higher than in the healthy population (1, 10, 93, 94). In a very recent study, Ashraf et al. reported that the prevalence of sleep disturbance reached 67.9% in a population of patients with solid malignancies. The complaint was mostly not addressed by attending oncologists (95). The wide reported incidence range probably reflects different populations and methodology, particularly questionnaires versus symptom reporting (13). Various diagnostic criteria are used, including broadly defined sleep problems per Common Terminology Criteria for Adverse Events (CTCAE), and, at the other end of the spectrum, the very detailed insomnia disorder (i.e. primary insomnia) definition provided by the Diagnostic and Statistical Manual of Mental Disorders (78, 96). Notably, the latter excludes medication-induced insomnia and is consequently less useful for the oncology practice.

There are several limitations of our meta-analysis. There is the possibility of underreporting the very common symptom present at baseline in many patients, and the fact that the severity and type of sleep disturbances may change over the course of cancer and therapy. Longitudinal evolution of insomnia in clinical trials can be assessed using formal quality of life (QoL) analysis using standard QoL questionnaires. Sleep disturbances are more prevalent in women and there is also a stronger association between insomnia and inflammation in females (7) but we have not been able to account for this fact in the present meta-analysis as gender-specific toxicity data were not available from published sources. Insomnia has been reported as an early symptom in autoimmune endocrine abnormalities in patients treated with CPI but we did not test this correlation in the present study (97). Insomnia is also linked to cognitive impairment (98). There are currently few reports assessing the cognitive sequelae of CPI therapy, and the topic remains an interesting research question for the future (99).

## Conclusion

Cancer immunotherapy using CPI is clearly associated with insomnia. The risk of insomnia as an adverse event was not significantly higher in patients treated with CPI compared to those receiving chemotherapy. AntiCTLA-4 agents are associated with higher incidence of insomnia compared to PD-1/PD-L1 inhibitors.

## Data availability statement

The original contributions presented in the study are included in the article/**supplementary material**. Further inquiries can be directed to the corresponding author.

## Author contributions

TB had the right to deal with all the data and was responsible for the decision to submit the manuscript for publication. IK, MK and TB had the data of all included clinical trials. TB, BB and KH retrieved the data. MK carried out the statistical analysis. LB extensively revised the manuscript and provided interpretation of the statistical methods and results. TB, IK and BB were responsible for checking and evaluating the quality of the collected data. All authors contributed to the article and approved the submitted version.

## Funding

Publication fees have been covered by unrestricted grant from Roche, Servier, AstraZeneca, and Bristol Myers Squibb. The funder was not involved in the study design, collection, analysis, interpretation of data, the writing of this article or the decision to submit it for publication.

## Conflict of interest

IK has received research support and honoraria from Roche, Bristol Myers Squibb, Merck Sharp Dohme, Merck, and Servier, all unrelated to the present paper. TB has received research support and honoraria from Roche, Bristol Myers Squibb, Merck Sharp Dohme, Merck, and AstraZeneca, all unrelated to the present paper.

The remaining authors declare that the research was conducted in the absence of any commercial or financial relationships that could be constructed as a potential conflict of interest.

## Publisher’s note

All claims expressed in this article are solely those of the authors and do not necessarily represent those of their affiliated organizations, or those of the publisher, the editors and the reviewers. Any product that may be evaluated in this article, or claim that may be made by its manufacturer, is not guaranteed or endorsed by the publisher.
