# Supplementary material for: Insomnia in patients treated with checkpoint inhibitors for cancer: A meta-analysis
Source: Front Oncol. 2022 Aug 2;12:946307. doi: 10.3389/fonc.2022.946307 (PMC9380599; doi:10.3389/fonc.2022.946307)
Supplement: Supplementary file 1 [file Table_1.docx]

**Supplementary Table 1.**

**List of studies.**

| **NCT** | **Author** | **Year** | **Number of participants** | **Study Name** | **Treatments** | **Blinding** | **Cancer** |
| --- | --- | --- | --- | --- | --- | --- | --- |
| NCT00094653 | Hodi et al. 2010 | 2010 | 676 | MDX010-20 | Ipilimumab + gp100 vs Ipilimumab vs gp100 | b | Melanoma |
| NCT00324155 | Robert et al. 2011 | 2011 | 502 |  | Ipilimumab + chemotherapy vs Placebo + Chemotherapy | b | Melanoma |
| NCT00861614 | Kwon et al. 2014 | 2014 | 799 | CA184-043 | Ipilimumab vs Placebo | b | Prostate |
| NCT01673867 | Borghaei et al. 2015 | 2015 | 582 | CheckMate 057 | Nivolumab vs Chemotherapy | o | Lung |
| NCT01642004 | Brahmer et al. 2015 | 2015 | 272 | CheckMate 017 | Nivolumab vs Chemotherapy | o | Lung |
| NCT01668784 | Motzer et al. 2015 | 2015 | 821 | CheckMate 025 | Nivolumab vs Everolimus | o | Renal |
| NCT01721772 | Robert et al. 2015 | 2015 | 418 | CheckMate 066 | Nivolumab vs Chemotherapy | b | Melanoma |
| NCT01905657 | Herbst et al. 2016 | 2016 | 1033 | KEYNOTE-010 | Pembrolizumab vs Chemotherapy | o | Lung |
| NCT01450761 | Reck et al. 2016 | 2016 | 954 |  | Ipilimumab + chemotherapy vs Placebo + chemotherapy | b | Lung |
| NCT02142738 | Reck et al. 2016 | 2016 | 305 | KEYNOTE-024 | Pembrolizumab vs Chemotherapy | b | Lung |
| NCT00636168 | Eggermont et al. 2016 | 2016 | 1211 | EORTC 18071 | Ipilimumab vs Placebo | b | Melanoma |
| NCT02105636 | Ferris et al. 2016 | 2016 | 361 | CheckMate 141 | Nivolumab vs Chemotherapy | o | Head and neck |
| NCT02041533 | Carbone et al. 2017 | 2017 | 541 | CheckMate 026 | Nivolumab vs Chemotherapy | o | Lung |
| NCT02125461 | Antonia et al. 2017 | 2017 | 713 | PACIFIC | Durvalumab vs Placebo | b | Lung |
| NCT02008227 | Rittmeyer et al. 2017 | 2017 | 1225 | OAK | Atezolizumab vs Chemotherapy | o | Lung |
| NCT01057810 | Beer et al. 2017 | 2017 | 602 | CA184-095 | Ipilimumab vs Placebo | b | Prostate |
| NCT02256436 | Bellmunt et al. 2017 | 2017 | 542 | KEYNOTE-045 | Pembrolizumab vs Chemotherapy | o | Urothelial |
| NCT01843374 | Maio et al. 2017 | 2017 | 571 | DETERMINE | Tremelimumab vs Placebo | b | Mesothelioma |
| NCT02395172 | Barlesi et al. 2018 | 2018 | 529 | JAVELIN Lung 200 | Avelumab vs Chemotherapy | o | Lung |
| NCT02578680 | Gandhi et al. 2018 | 2018 | 616 | KEYNOTE-189 | Pembrolizumab + chemotherapy vs Placebo + chemotherapy | b | Lung |
| NCT02763579 | Horn et al. 2018 | 2018 | 403 | IMpower133 | Atezolizumab + chemotherapy vs Placebo + chemotherapy | b | Lung |
| NCT01721746 | Larkin et al. 2018 | 2018 | 405 | CheckMate 037 | Nivolumab vs Chemotherapy | o | Melanoma |
| NCT02231749 | Motzer et al. 2018 | 2018 | 847 | CheckMate 214 | Nivolumab + ipilimumab vs Sunitinib | o | Renal |
| NCT02775435 | Paz-Ares et al. 2018 | 2018 | 559 | KEYNOTE-407 | Pembrolizumab + chemotherapy vs Placebo + chemotherapy | b | Lung |
| NCT02366143 | Socinski et al. 2018 | 2018 | 1202 | IMpower150 | Atezolizumab + bevacizumab + chemotherapy vs Bevacizumab + chemotherapy | o | Lung |
| NCT02302807 | Powles et al. 2018 | 2018 | 931 | IMvigor211 | Atezolizumab vs Chemotherapy | o | Urothelial |
| NCT02370498 | Shitara et al. 2018 | 2018 | 395 | KEYNOTE-061 | Pembrolizumab vs Chemotherapy | o | Gastric or GEJ |
| NCT02425891 | Schmid et al, 2018 | 2018 | 902 | IMpassion130 | Atezolizumab + chemotherapy vs Placebo + Chemotherapy | b | Breast |
| NCT02763579 | Horn et al, 2018 | 2018 | 403 | IMpower133 | Atezolizumab + chemotherapy vs Placebo + chemotherapy | b | Lung |
| NCT02220894 | Mok et al. 2019 | 2019 | 1274 | KEYNOTE-042 | Pembrolizumab vs Chemotherapy | o | Lung |
| NCT02853331 | Rini, Plimack, et al. 2019 | 2019 | 861 | KEYNOTE-426 | Pembrolizumab + axitinib vs Sunitinib | o | Renal |
| NCT02367781 | West et al. 2019 | 2019 | 679 | IMpower130 | Atezolizumab + chemotherapy vs Chemotherapy | o | Lung |
| NCT02420821 | Rini, Powles, et al. 2019 | 2019 | 915 | IMmotion151 | Atezolizumab + Bevacizumab vs Sunitinib | o | Renal |
| NCT03043872 | Paz-Ares et al. 2019 | 2019 | 988 | CASPIAN | Durvalumab + tremelimumab + chemotherapy vs Durvalumab + chemotherapy vs Chemotherapy | o | Lung |
| NCT02252042 | Cohen et al. 2019 | 2019 | 495 | KEYNOTE-040 | Pembrolizumab vs Chemotherapy | o | Head and neck |
| NCT02788279 | Eng et al. 2019 | 2019 | 363 | IMblaze370 | Atezolizumab + cobimetinib vs Atezolizumab vs Regorafenib | o | Colorectal |
| NCT02358031 | Burtness et al, 2019 | 2019 | 882 | Keynote-048 | Pembrolizumab + chemotherapy vs cetuximab + chemotherapy | o | Head and neck |
| NCT02613507 | Wu et al, 2019 | 2019 | 639 | CheckMate 078 | Nivolumab vs Chemotherapy | o | Lung |
| NCT02908672 | Gutzmer et al. 2020 | 2020 |  | IMspire150 | Atezolizumab + Cobimetinib + Vemurafenib vs Placebo + Cobimetinib + Vemurafenib | b | Melanoma |
| NCT02367794 | Jotte et al, 2020 | 2020 | 1021 | IMpower131 | Atezolizumab + chemotherapy vs Chemotherapy | o | Lung |
| NCT02369874 | Ferris et al, 2020 | 2020 | 736 | EAGLE | Durvalumab + tremelimumab vs durvalumab vs Chemotherapy | o | Head and neck |
| NCT02409342 | Herbst et al, 2020 | 2020 | 572 | IMpower110 | Atezolizumab vs Chemotherapy | o | Lung |
| NCT02453282 | Rizvi et al, 2020 | 2020 | 1118 | MYSTIC | Durvalumab + tremelimumab vs Tremelimumab vs Chemotherapy | o | Lung |
| NCT02516241 | Powles et al. 2020 | 2020 | 1126 | DANUBE | Durvalumab + tremelimumab vs Tremelimumab vs Chemotherapy | o | Urothelial |
| NCT02564263 | Kojima et al., 2020 | 2020 | 628 | KEYNOTE-181 | Pembrolizumab vs Chemotherapy | o | Oesophagus or GEJ |
| NCT02603432 | Powles et al, 2020 | 2020 | 700 | JAVELIN Bladder 100 | Avelumab vs Observation | o | Urothelial |
| NCT02702401 | Finn et al., 2020 | 2020 | 413 | KEYNOTE-240 | Pembrolizumab vs Best supportive care | o | Hepatocellular |
| NCT03066778 | Rudin et al., 2020 | 2020 | 453 | KEYNOTE-604 | Pembrolizumab + chemotherapy vs Placebo + chemotherapy | b | Lung |
| NCT03197935 | Mittendorf et al. 2020 | 2020 | 333 | IMpassion031 | Atezolizumab + chemotherapy vs Placebo + chemotherapy | b | Breast |
| NCT02538666 | Owonikoko et al, 2021 | 2021 | 1212 | CheckMate 451 | Nivolumab+ipilimumab vs Nivolumab vs Placebo | b | Lung |
| NCT02555657 | Winer et al, 2021 | 2021 | 622 | KEYNOTE-119 | Pembrolizumab vs Chemotherapy | o | Breast |
| NCT02853305 | Powles et al, 2021 | 2021 | 1010 | KEYNOTE-361 | Pembrolizumab chemotherapy vs Chemotherapy | o | Urothelial |
| NCT02899299 | Baas et al., 2021 | 2021 | 605 | CheckMate 743 | Nivolumab + ipilimumab vs Chemotherapy | o | Mesothelioma |
| NCT03215706 | Paz-Ares et al, 2021 | 2021 | 719 | CheckMate 9LA | Ipilimumab + nivolumab + chemotherapy vs Chemotherapy | o | Lung |
| NCT02811861 | Motzer et al, 2021 | 2021 | 1069 | CLEAR | Pembrolizumab + lenvatinib versus Sunitinib versus Lenvatinib + everolimus | o | Renal |

GEJ, gastro-oesphageal junction; b, double-blinded; o, open-label
